# Van der Waals Heterostructures With Built‐In Mie Resonances For Polarization‐Sensitive Photodetection

**DOI:** 10.1002/advs.202207022

**Published:** 2023-01-22

**Authors:** Jiahao Yan, Xinzhu Yang, Xinyue Liu, Chun Du, Fei Qin, Mengmeng Yang, Zhaoqiang Zheng, Jingbo Li

**Affiliations:** ^1^ Institute of Nanophotonics Jinan University Guangzhou 511443 P. R. China; ^2^ Guangdong Provincial Key Laboratory of Optical Fiber Sensing and Communications Institute of Photonics Technology Jinan University Guangzhou 511443 P. R. China; ^3^ Guangdong Provincial Key Laboratory of Information Photonics Technology School of Materials and Energy Guangdong University of Technology Guangzhou 510006 P. R. China; ^4^ Institute of Semiconductors South China Normal University Guangzhou 510631 P. R. China

**Keywords:** Mie resonances, nanopatterned heterostructures, polarization‐sensitive photodetection, ReS_2_, WSe_2_

## Abstract

Few‐layer transition metal dichalcogenides (TMDs) and their combination as van der Waals heterostructures provide a promising platform for high‐performance optoelectronic devices. However, the ultrathin thickness of TMD flakes limits efficient light trapping and absorption, which triggers the hybrid construction with optical resonant cavities for enhanced light absorption. The optical structure enriched photodetectors can also be wavelength‐ and polarization‐sensitive but require complicated fabrication. Herein, a new‐type TMD‐based photodetector embedded with nanoslits is proposed to enhance light trapping. Taking ReS_2_ as an example, strong anisotropic Mie‐type optical responses arising from the intrinsic in‐plane anisotropy and nanoslit‐enhanced anisotropy are discovered. Owing to the nanoslit‐enhanced optical resonances and band engineering, excellent photodetection performances are demonstrated with high responsivity of 27 A W^−1^ and short rise/decay times of 3.7/3.7 ms. More importantly, through controlling the angle between the nanoslit orientation and the polarization direction to excite different resonant modes, polarization‐sensitive photodetectors with anisotropy ratios from 5.9 to 12.6 can be achieved, representing one of the most polarization‐sensitive TMD‐based photodetectors. The depth and orientation of nanoslits are demonstrated crucial for optimizing the anisotropy ratio. The findings bring an effective scheme to construct high‐performance and polarization‐sensitive photodetectors.

## Graphical abstract

In this work, van der Waals heterostructures with built‐in nanoslit arrays are demonstrated for high‐performance and polarization‐sensitive photodetection. Owing to the nanoslit‐enhanced anisotropic Mie resonances and band engineering, excellent photodetection performances are demonstrated. Through controlling the angle between the nanoslit orientation and the polarization direction to excite different optical resonant modes, the anisotropy ratios for polarization‐sensitive photodetection are controllable.

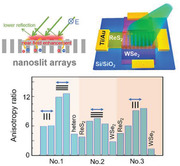

## 1 Introduction

The vertical stacking of transition metal dichalcogenides (TMDs) forming as van der Waals (vdW) heterostructures shows intriguing optoelectronic properties.^[^ ^1^, ^2^, ^3^, ^4^ ^]^ Especially, the type‐II alignment of TMDs such as WS_2_/MoS_2_ and WSe_2_/MoS_2_ can achieve efficient electron–hole separation and benefit the applications of photodetectors, photovoltaic cells, and light emitting diodes.^[^ ^5^, ^6^, ^7^, ^8^ ^]^ However, the low absorptivity of atomically layered 2D TMDs limits the improvement of photoresponsivity. On the other hand, the detection of polarized light based on TMDs remains a challenge. Achieving the detection of polarized light is significant for polarization imaging, target tracking, and remote sensing.^[^ ^9^ ^]^ Fortunately, some TMDs such as ReS_2_ with intrinsic in‐plane anisotropy^[^ ^10^, ^11^, ^12^ ^]^ enrich the devices for polarization‐sensitive detection.^[^ ^13^, ^14^, ^15^, ^16^ ^]^ Utilizing intrinsic anisotropy to realize polarization detection is insensitive; so, this limitation triggers the combination of TMDs with metallic plasmonic structures^[^ ^17^, ^18^, ^19^ ^]^ or dielectric Mie‐type structures^[^ ^20^, ^21^, ^22^, ^23^ ^]^ to enhance light–matter interactions.^[^ ^24^, ^25^ ^]^ By tuning the resonant modes through structural design, optical nanostructures facilitate wavelength‐dependent or polarization‐sensitive photodetection of TMDs.^[^ ^26^, ^27^, ^28^, ^29^, ^30^ ^]^ However, these hybrid devices require sophisticated fabrication process or time‐consuming assembly techniques.

To solve the above problems, it is important to propose TMD‐based photodetectors with built‐in optical functions. Inspired by silicon solar cells enhanced by nanostructured surfaces,^[^ ^31^, ^32^, ^33^ ^]^ it is conceivable that nanopatterned TMD flakes are beneficial for efficient light‐harvesting and photodetection. Notably, most TMDs possess high refractive indices which may be even higher than those of silicon, which can generate strong Mie resonances for subwavelength light confinement and near‐field enhancement. Furthermore, TMD flakes with out‐of‐plane and in‐plane anisotropic refractive indices^[^ ^34^, ^35^ ^]^ offer more degrees of freedom for light manipulation. Previous studies have focused on their optical effects and the intrinsic Mie‐exciton coupling.^[^ ^36^, ^37^, ^38^, ^39^, ^40^, ^41^ ^]^ How to take advantages of strong optical resonances of nanopatterned TMDs to build photodetectors remains unresolved.

Here, we first propose TMD‐based photodetectors with built‐in nanoslits to reinforce optical functionality. The synergistic effect of optical Mie resonance and optoelectronic properties facilitates high‐performance and polarization‐sensitive photodetection. Through the spectral analysis of single ReS_2_ nanoslits and arrays of ReS_2_ nanoslits, strong anisotropic Mie resonances are witnessed owing to the intrinsic in‐plane anisotropy and the slit‐enhanced anisotropy. To explore how nanoslit arrays affect the photodetection performance, we fabricated patterned WSe_2_/ReS_2_ heterostructures with three configurations. For unpatterned WSe_2_/ReS_2_ heterostructures, strong suppression of the photoluminescence (PL) intensity is observed due to the type‐II alignment. However, for the patterned WSe_2_/ReS_2_ heterostructures, the PL signal is re‐enhanced which indicates that light trapping and optical resonance play important roles.

Through photocurrent measurements of nanopatterned WSe_2_/ReS_2_ heterostructures, we recorded high responsivity (*R*) of 27.3 A W^−1^ and short rise/decay times of 3.7/3.7 ms. Compared with unpatterned heterostructures, the resonance‐enhanced light trapping compensates for the impaired electron–hole separation due to focused ion beam (FIB)‐induced defects and leads to comparable responsivity. How band engineering affects photodetection performance is investigated in three configurations of WSe_2_/ReS_2_ heterostructures. Multi‐points measurements of different regions using focused laser beam demonstrate that the Mie resonance of the ReS_2_ nanoslit can enhance the photocurrent fivefold than unpatterned area. Moreover, through scanning the laser beam with varied polarization onto devices, the photocurrent anisotropy ratio (*I_b_*_‐axis_/*I_a_*_‐axis_) can be tuned from 1.4 to 12.6. The consistent direction of nanoslit, *b*‐axis, and polarization yielded the highest anisotropy ratio of 12.6, which is the best performance for a TMD‐based photodetector. Further optimization of structure parameters may lead to better performance. The proposed photodetection platforms based on nano‐patterned TMDs will inspire the design of 2D material‐based optoelectronic devices with more built‐in optical functions.

## 2 Results and Discussion

**Figure 1:**
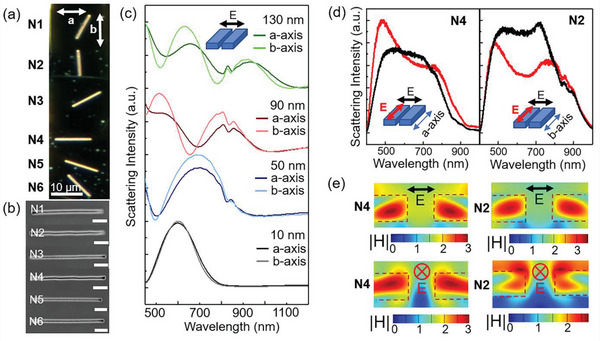
Anisotropic optical resonances of single ReS_2_ nanoslits. a) Dark‐field optical images and b) SEM images of ReS_2_ nanoslits with varied orientations. The directions of *a*‐ and *b*‐axes are marked by arrows. Scale bar: 500 nm. c) Simulated scattering spectra of nanoslits patterned along *a*‐ or *b*‐axis on ReS_2_ flakes with varied thicknesses. d) Measured scattering spectra of nanoslits under linearly polarized light. The embedded diagram shows the directions of polarization and nanoslit. e) Near‐field magnetic field distributions (|H|) of ReS_2_ nanoslits at the wavelengths where the magnetic field reaches a maximum.

Optical resonant structures made of TMDs show spectral tuning capabilities owing to their high refractive indices and excitonic transitions. Among them, ReS_2_ is more special because of the in‐plane anisotropic refractive index. The motivation of using ReS_2_ can be further explained by the wavelength‐dependent refractive index (*n*) and extinction coefficient (*k*) shown in Figure S1, Supporting Information. *n*_ReS2_ along both *a*‐ and *b*‐axes is comparable to that of silicon, which belongs to high *n* dielectric materials. Although higher *k*_ReS2_ than silicon may induce larger absorption, this side effect is beneficial for light harvesting and photodetection. More importantly, *n*_ReS2_ and *k*_ReS2_ show noticeable differences along *a*‐ and *b*‐axes, which makes ReS_2_ possible for constructing anisotropic photodetectors. ReS_2_ flakes are nanopatterned into nanoslits through FIB milling. The thicknesses of the ReS_2_ flakes are carefully controlled during exfoliation and transfer processes to generate different resonant modes. Owing to the distorted 1T highly‐anisotropic in‐plane structure of ReS_2_ flakes, ReS_2_ crystals can be easily cleaved along the *b*‐axis (the Re—Re atomic chain direction). Therefore, the mechanical exfoliation process produces a ribbon‐shaped crystal, and the *b*‐axis of ReS_2_ can be determined simply through checking the long edge.^[^ ^11^, ^12^ ^]^ As an example, **Figure** **1**a shows a typical ReS_2_ flake with a thickness of 120 nm and fabricated nanoslits N1‐N6 with 0–360^o^ orientation. The scanning electron microscope (SEM) image (Figure 1b) shows that the width of nanoslits is ≈150 nm. Spectral tailoring of the scattering from a single ReS_2_ nanoslit is achieved by varying the thickness as shown in Figure 1c, which also reflects the change of resonant mode. For a ReS_2_ nanoslit with a thickness of 10 nm, whether the nanoslit is along *a*‐axis or *b*‐axis has little influence on the scattering spectrum, indicating that the ReS_2_ flake is too thin to support Mie‐type resonant modes. When further increasing the thickness to 50, 90, and 130 nm, clear differences between the nanoslits along two orthogonal axes are witnessed. The main resonant peak experiences red‐shift with the increase of thickness because of the increasing optical path difference.

The above analysis is under the polarization direction perpendicular to the nanoslit. To further investigate polarization‐dependent scattering, we focus on two typical nanoslits along the *a*‐axis and *b*‐axis of ReS_2_, named as N4 and N2, respectively, in Figure 1a. For the nanoslit N4 (Figure 1d), two distinct peaks are observed at 500 and 800 nm under the excitation of parallel polarization, while only one broad peak located near 600 nm exists under vertically polarized light. For the nanoslit along *b*‐axis (N2 in Figure 1d), the variation trend is similar but the scattering intensity is stronger than nanoslit N4 for wavelengths greater than 600 nm. The simulated results in Figure S2, Supporting Information, show a similar trend, and the deviations between experiments and simulations mainly come from the differences of refractive indices caused by the FIB‐induced amorphization. The cross‐sectional profiles of the maximum magnetic field are shown in Figure 1e, and the detailed magnetic and electric field profiles at more wavelengths are presented in Figure S2, Supporting Information. Obviously, the near‐field profiles under vertical and parallel polarizations exhibit distinct differences, which induce differences in the scattering spectra. The circular magnetic field distributed on both sides of the air gap indicates the generation of Mie‐type resonances. In Figure S2, Supporting Information, the electric field distributed at the edge under vertical polarization and distributed inside ReS_2_ under horizontal polarization clearly indicates the strong influence of polarization direction. Moreover, the different distributions of electromagnetic fields will determine their contribution to the photodetection performance. For nanoslits with the same polarization but with different crystal orientations, the field profiles also show obvious differences, further confirming the anisotropic optical resonance.

**Figure 2:**
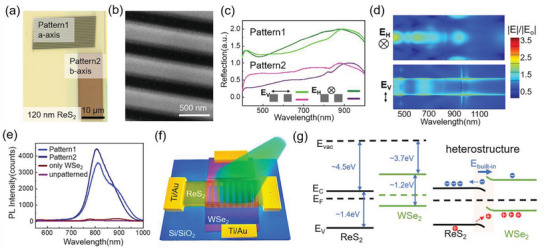
Optical properties of ReS_2_ nanoslit arrays and WSe_2_/ReS_2_ heterostructures. a) The bright‐field optical image and b) SEM image of ReS_2_ nanoslit arrays. The orientation of the nanoslits is along *a*‐axis (*b*‐axis) in Pattern 1 (2). c) Measured reflection spectra of the nanoslit array on 120 nm ReS_2_ under horizontal (*E*_H_) or vertical (*E*_V_) polarization. d) Wavelength‐dependent electric field distributions along the top surface of the nanoslit (Pattern 1) under two polarization directions. e) PL spectra of few‐layer WSe_2_ on the Si/SiO_2_ substrate, the unpatterned ReS_2_, and the patterned ReS_2_ (Patterns 1 and 2). f) Configuration of WSe_2_/ReS_2_ heterostructures. g) Energy band diagrams of few‐layer ReS_2_ (unpatterned) and WSe_2_ before and after contact.

For optoelectronic applications, ReS_2_ nanoslit arrays were fabricated to enlarge the light‐harvesting area. The optical image in **Figure** **2**a shows a typical nanoslit array along the *a*‐ or *b*‐axis on a ReS_2_ flake, and the atomic force microscope (AFM) measurement shown in Figure S3, Supporting Information, gives the exact thickness: 120 nm. Different colors of arrays along two directions clearly indicate that the reflectance spectra are different. The morphology of a typical nanoslit array is revealed by the SEM image shown in Figure 2b. The polarization‐dependent reflectance spectra of the two arrays are measured as shown in Figure 2c,d. Obviously, the crystal orientation exhibits strong influence on the reflectance spectra. Especially under vertical polarization, the reflectance of the nanoslit along *b*‐axis is three times higher than that along *a*‐axis for wavelengths less than 800 nm. Nanoslit arrays are also patterned on a thinner ReS_2_ flake (28 nm) as shown in Figure S4, Supporting Information. Similar to the study of single nanoslits, thin nanoslit arrays are not able to generate Mie‐type resonances. Therefore, as shown in Figure S4b, Supporting Information, both the crystal orientation‐dependent and polarization‐dependent reflections show inconspicuous differences. The wavelength‐dependent field distributions on the top surface are further simulated and presented in Figure 2d; Figure S4c–e, Supporting Information, to indicate near‐field absorption of Patterns 1–4. Obviously, the increased thickness enriches the Mie‐type resonant modes, so the wavelength‐dependent near‐field enhancement in Patterns 1 and 2 is more complex than in Patterns 3 and 4. The spatial distribution is different under two polarizations: the electric field distributes in the middle of nanoslit under horizontal polarization, while the electric field distributes on the edges under vertical polarization. Through the comparison of nanoslit arrays along different axes of ReS_2_, we find that the angle between crystal orientation and polarization direction has less influence than the angle between nanoslit orientation and polarization direction. The wavelength‐dependent near‐field distributions of nanoslit arrays along *a*‐axis and *b*‐axis on 120 nm ReS_2_ (Figure 2d; Figure S4c, Supporting Information) have larger contrast than those on 28 nm ReS_2_ (Figure S4d,e, Supporting Information), indicating much stronger anisotropic Mie resonances, which is similar to the study on single nanoslits in Figure 1c,d. The wavelengths where the electric field is enhanced correspond to the wavelengths where the reflectivity is suppressed based on Figure 2c,d.

To examine the light trapping effect of nanoslit arrays, the heterostructures are assembled by transferring few‐layer WSe_2_ on the patterned ReS_2_. The optical images in Figure S5, Supporting Information, show the presence of few‐layer WSe_2_ on ReS_2_ nanoslit arrays. PL from different regions (Figure 2e; Figure S5a, Supporting Information) are studied to check the generation and separation of electron–hole pairs and how the optical resonances influence those processes. Although ReS_2_ flakes are direct‐band semiconductors which are independent of thicknesses, the quantum yield of PL is much lower than that of few‐layer WSe_2_.^[^ ^13^ ^]^ Therefore, only the PL of WSe_2_ dominates. Owing to the type‐II alignment between WSe_2_ and ReS_2_, the effective separation of photogenerated electrons and holes greatly suppresses the PL intensity, and the intensity ratio (*I*_WSe2/ReS2‐unpatterned_/*I*_WSe2_) is less than 30% at the excitonic peak (Figure 2e). On the contrary, the PL intensity is further enhanced with the intensity ratios (*I*_WSe2/ReS2‐patterned_/*I*_WSe2_) more than ten on both Patterns 1 and 2. Apart from etched regions that weaken the charge‐separation effect, the optical resonances play a significant role regarding the PL enhancement. For a few‐layer WSe_2_ on the 28 nm‐ReS_2_ flake (Figure S5a, Supporting Information), the quenching of PL intensity is still observed, but the PL enhancement effect on nanoslits is much weaker than that of 120 nm ReS_2_ flakes. As the excitonic emission of few‐layer WSe_2_ exhibits two peaks: excitons at 776 nm and trions at 882 nm, the detailed PL intensity ratios as well as the standard deviations are presented in Figure S5d, Supporting Information.

Through the above analysis on PL manipulation, we can find the coexistence of efficient charge separation and strong optical resonance. ReS_2_ nanoslit arrays fabricated by FIB milling increase the surface area and Ga‐ion induced defects,^[^ ^32^, ^42^ ^]^ which enhance the photocarrier recombination and weaken the charge separation. If the charge recombination effect dominates, the PL intensity of the patterned WSe_2_/ReS_2_ should be equal to that of the WSe_2_‐only region. However, as discussed above, the intensity ratios (*I*_WSe2/ReS2‐patterned_/*I*_WSe2_) can exceed 10, which indicates that the increased absorption and emission rate owing to the optical resonances of nanoslit arrays dominate the PL manipulation. Both the increase in absorption and emission rate originate from the strong near‐field enhancements,^[^ ^43^ ^]^ which also play an important role on the improvement of photodetection. Therefore, we can conclude that the advantages of optical nanostructures surpass their side effect while bringing new functionalities to polarization detection. Photodetectors based on these heterostructures are constructed as schematically shown in Figure 2f. For unpatterned heterostructures, the bandstructure of few‐layer WSe_2_ and ReS_2_ shows a typical type‐II alignment.^[^ ^13^, ^14^ ^]^ After contact, a built‐in electric field at the hetero‐interface can be established that benefits the charge separation and current rectification. In the next step, we will explore how nanoslits affect photodetection from the aspects of optical resonance and energy band engineering.

Three strategies are used to build nanoslit‐enhanced WSe_2_/ReS_2_ photodetectors, named bottom‐patterned (No.1), top‐patterned (No.2), and full‐patterned (No.3). The thickness of each layer is determined from the AFM data in Figures S3 and S6, Supporting Information. Except for the bottom‐patterned photodetector which cannot be measured before patterning, we first study the photoresponses of unpatterned Nos. 2 and 3 photodetectors (Figure S7a, Supporting Information). To quantify the photodetection performance of the two devices, *I–V* curves under 405 and 532 nm laser incidence with different intensities are presented in Figure S7b, Supporting Information. The photocurrent of No. 2 is much lower than that of No. 3 because of smaller photosensitive area. All *I–V* curves have similar variation trends, and the open‐circuit voltages (*V*_OC_) are greater than zero, indicating the built‐in potential of heterostructures. The photoswitching performance in Figure S7c, Supporting Information, indicates rise/decay times of 13.1/17.1 ms, but the photocurrent fluctuates upon illumination. As we know, high‐speed photoswitching is attributed to the effective separation of electron–hole pairs inside heterostructures. Compared with the regions with only WSe_2_ and ReS_2_, the heterojunction also shows a more pronounced diode‐like current rectification behavior, as shown in the *I–V* curves in Figure S8, Supporting Information. In Figures S9 and S10, Supporting Information, detailed *I–V* measurements of only WSe_2_ and ReS_2_ regions under different light intensities are given. Only ReS_2_ shows comparable photocurrent but much larger dark current than the heterojunction. Moreover, the photocurrent of only WSe_2_ region is much smaller than that of the heterojunction region. The above analysis demonstrates the advantage that the heterojunctions can significantly increase the photocurrent.

**Figure 3:**
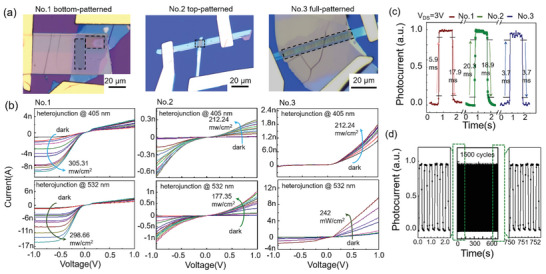
Photodetection performances of patterned WSe_2_/ReS_2_ heterostructures. a) Optical images of three typical hetero‐devices: No.1 bottom‐patterned, No.2 top‐patterned, and No.3 full‐patterned. b) *I–V* characteristics of the patterned WSe_2_/ReS_2_ photodetectors under light irradiation (405 and 532 nm) with various intensities. c) Temporal photoresponse for a single photoswitching cycle of patterned photodetectors No. 1–3. d) Time‐resolved photoresponse of device No. 3 (patterned) under periodic 532 nm light for 1500 cycles.

In **Figure** **3**a, optical images of nanoslit‐enhanced WSe_2_/ReS_2_ photodetectors fabricated via three strategies are presented. *I–V* curves of the three devices under 405 and 532 nm illumination with different intensities are presented in Figure 3b. Compared to the unpatterned device, the Photodetector No. 2 shows similar *I–V* characteristics and higher photocurrent under 532 nm light. For the No. 3 photodetector, the *I–V* curves after patterning show a different trend compared with the unpatterned device. Although the photocurrent decreases after etching, the current rectification effect changes strongly. The forward‐to‐reverse bias current ratio is 19 times larger than that of unpatterned device. This difference arises from the altered band alignment and charge transfer owing to the Ga‐ion injection into heterointerfaces.^[^ ^44^ ^]^ In addition, the photocurrents of all samples under 532 nm light are obviously higher than those under 405 nm light, which is different from the comparable photocurrents of unpatterned samples. This indicates that the optical resonance of nanoslits strongly enhances the photocurrent at 532 nm incidence, superimposed on the initial response due to the intrinsic absorption of heterostructures. To check the ability of high‐speed applications of the fabricated photodetectors, temporal photoresponses of the three devices are shown in Figure 3c, labeled by rise/decay times. The photocurrent can rise rapidly to a stable value after illumination and fall quickly without illumination. As expected, the patterned photodetectors can obtain 3.5 times faster response time than the best performance of the unpatterned heterostructure. These results further confirm the better electron–hole separation of patterned devices. We also test the reliability and stability of the patterned photodetector in Figure 3d. After 1500 photoswitching cycles, the periodically repeated photocurrent has no obvious deterioration.

**Figure 4:**
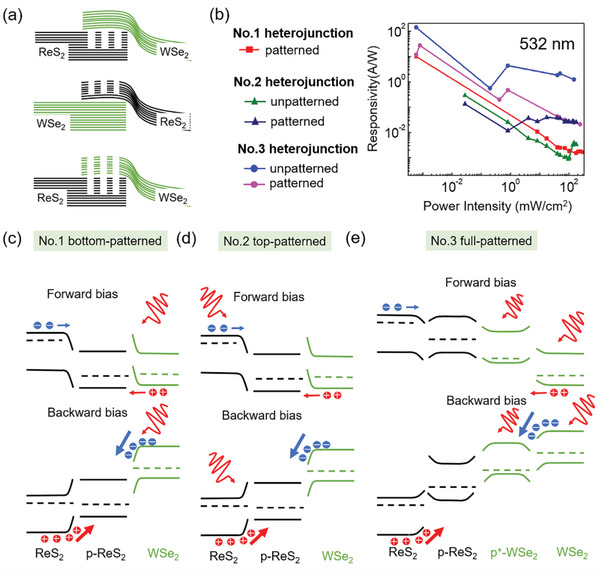
Analysis of patterned WSe_2_/ReS_2_ photodetectors. a) Schematic of three configurations of patterned WSe_2_/ReS_2_ photodetectors. b) Responsivity of unpatterned and patterned heterostructures (Nos. 1–3) calculated from the photocurrent measurements. c‐e) Energy band diagrams and corresponding carrier transfer processes of patterned heterostructures (Nos. 1–3) under illumination.

To better understand the three configurations of the patterned WSe_2_/ReS_2_ photodetector, the cross‐sectional sketches are presented in **Figure** **4**a. Responsivity defined as $R=\frac{I_{\mathrm{light}}-I_{\mathrm{dark}}}{P_{\mathrm{laser}}}$ is an important parameter that evaluates the photodetection performance, where *I*_light_ and *I*_dark_ are the device current with and without illumination, respectively, and *P*_laser_ is the incident light power. The variation of *R* with *P*_laser_ (532 nm) for different heterostructures with or without nanoslits is presented in Figure 4b. Generally, *R* decreases as a function of *P*_laser_, and some fluctuations arise from the formation of defects and charged impurities and the recombination processes of electron–hole pairs.^[^ ^45^, ^46^, ^47^ ^]^ For the Photodetector No.1 with bottom patterns, *R* can reach 10.0 A W^−1^, comparable to the best performance of conventional WSe_2_/ReS_2_ heterostructures.^[^ ^13^ ^]^ For the Photodetector No.2, the maximum *R* of unpatterned and patterned heterojunction is 0.29 and 0.13 A W^−1^, respectively. Compared with the No.1 photodetector, the much smaller photosensitive area as well as the smaller patterned area lead to a weaker photoresponse. Although *R* of the patterned device is smaller than that of unpatterned device at *P*_laser_ < 1 mW cm^−2^, it can be an order of magnitude higher than that of the unpatterned device at *P*_laser_ > 1 mW cm^−2^, demonstrating the obvious Mie resonance‐enhanced photoresponse under strong light irradiation. For the No. 3 photodetector, the maximum *R* of heterojunction is 140.3 A W^−1^ for the unpatterned device and 27.3 A W^−1^ for the patterned device. It can be seen that photoresponse of the unpatterned device is more significant than that of the patterned device at all laser intensities, implying that full‐patterned heterostructures do harm the photodetection performance through enhancing photocarrier recombination. The variation of *R* versus *P*_laser_ (405 nm) is also presented as shown in Figure S11, Supporting Information, where *R* of unpatterned and patterned devices are also comparable over certain laser intensity ranges. Especially at *P*_laser_ = 1.37 mW cm^−2^, *R* of the patterned device is three times larger than that of the unpatterned device. As discussed above, the optical resonance of nanoslits enhances the photocurrent; however, more defects and larger surface area lead to the carrier trapping that limits the separation process. Therefore, there is a trade‐off between additional optical functionality and overall responsivity. Fortunately, the enhancement factors from optical resonances are able to surpass the negative effects through further structural optimization.

As explained above, the impact of built‐in nanoslits embodies on light trapping and band engineering. We first explore the band engineering of the three configurations in depth, and the energy band diagrams in Figure 4c–e can help explain the photodetection performance. For unpatterned WSe_2_/ReS_2_ heterostructures, the built‐in electric field is overcome under forward bias (*V*_WSe2_ > *V*_ReS2_) as shown in Figure 2g, which produces a positive *V*_OC_ on *I–V* curves. Moreover, majority carriers (electrons of ReS_2_ and holes of WSe_2_) dominate the photoresponse when the valence band of ReS_2_ is higher than that of WSe_2_. Under backward bias (*V*_WSe2_ < *V*_ReS2_), the photo‐generated electrons of WSe_2_ and holes of ReS_2_ dominate the photoresponse. More effective electron–hole separation under backward bias facilitates much more obvious increase of photocurrents as indicated in Figure S7, Supporting Information. For patterned WSe_2_/ReS_2_ heterostructures, considerable Ga‐ion implantation occurs in the patterned region, which changes n‐type ReS_2_ to p‐type or p‐type WSe_2_ to p+‐type WSe_2_.^[^ ^44^ ^]^ For the No. 1 heterojunction with bottom patterning (Figure 4c), the electron–hole transportation is similar to unpatterned heterojunctions. Except under forward bias, the existence of additional energy bands creates potential barriers to trap free‐carriers. For the No. 2 heterojunction (Figure 4d), the top pattern has less influence on the carrier transportation under forward bias as shown in Figure 3; Figure S7, Supporting Information, because the laser illumination is directly applied on nanoslits which strongly enhances electron–hole generation. For the full‐patterned heterojunction (No. 3), the entire junction can be regarded as p‐WSe_2_/p+‐WSe_2_/p‐ReS_2_/n‐ReS_2_, as shown in Figure 4e, due to the FIB‐induced implantation on both layers. Without illumination, the No.3 heterojunction shows much larger current under forward bias than reverse bias due to the obvious majority carrier transportation. Actually, the efficiency of electron‐hole transportation is comparable under forward and backward bias with light illumination (Figure 3b). Similar to the case of the No. 2 heterojunction, direct irradiation on nanoslits strongly enhances the electron–hole generation, thereby compensating for the photogenerated carriers under forward bias.

**Figure 5:**
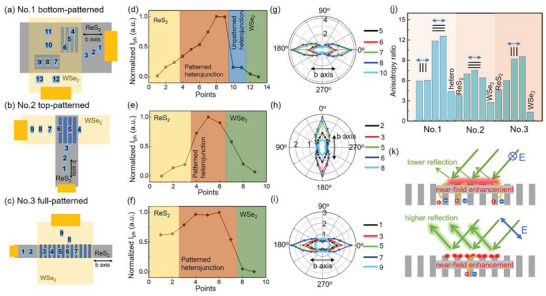
Mechanisms of nanoslit‐enhanced and polarization‐sensitive photodetection. a–c) Schematics showing the configurations of hetero‐devices and the locations of focused laser spots. d–f) Location‐dependent normalized photocurrents at *V*_ds_ = 2 V and *P*_laser_ = 72 mW cm^−2^. g–i) Polarization‐angle‐dependent normalized photocurrents measured from the locations marked in (a–c). j) A histogram showing the anisotropy ratios under all situations for the three devices (Nos.1–3). “ReS_2_,” “WSe_2_,” and “hetero” are used to denote the performances of ReS_2_‐only regions, WSe_2_‐only regions, and unpatterned heterojunctions, respectively. Blue arrows and black lines show the directions of crystal orientation and nanoslit orientation, respectively. k) Schematics to explain the optical resonances under horizontal and vertical polarizations and their contributions to electron–hole generation.

The above studies on energy band engineering and electron transport are the overall photoresponse excited by laser illumination covering the whole device. To further analyze the photodetection performance contributed by optical resonances, we measure the photocurrents at multiple locations in the patterned devices (Nos.1–3) using a focused laser beam with a diameter of 10 µm. The measured regions include only WSe_2_, only ReS_2_, unpatterned WSe_2_/ReS_2_, and patterned WSe_2_/ReS_2_ illustrated by simple schematic diagrams in **Figure** **5**a–c. The location‐dependent photocurrents are concluded in Figure 5d–f, and the detailed *I–V* curves are presented in Figure S12, Supporting Information. Obviously, the patterned heterojunctions show considerable photocurrent enhancement compared with the regions of only ReS_2_ and WSe_2_. This demonstrates that the WSe_2_/ReS_2_ heterojunction with patterning is ideal for enhancing photoresponse. Especially for the No. 2 device, the photocurrent excited at the patterned heterojunction is 70 times higher than that at the ReS_2_ region. Moreover, for the No. 1 device (Figure 5d), the patterned heterojunction exhibits fivefold larger photocurrent than unpatterned heterojunction.

The optoelectronic measurements above are performed under unpolarized light incidence. To examine how the optical resonance of nanoslits benefits the polarization‐sensitive photodetection, we further measure the photocurrent of different regions using focused laser beam (*λ* = 532 nm) with varied polarization directions. The polarization direction is changed from 0^°^ to 360^°^ in 15^°^ increments, and the polar plots of normalized photocurrents are shown in Figure 5g–i. The 0^°^ polarization direction is defined as the *b*‐axis direction of ReS_2_ flakes as illustrated in Figure 5a–c. In Figure 5g, we find obvious anisotropy of photocurrent also from the unpatterned WSe_2_/ReS_2_ heterojunction with an anisotropy ratio of 4.3, which originates from the intrinsic anisotropy of ReS_2_ crystal. The polarized light along *b*‐axis can generate larger photocurrents than that along *a*‐axis. The degree of anisotropy can be further enhanced on patterned heterojunction owing to the optical resonance of nanoslits. Interestingly, for the polarization direction along *b*‐axis, the nanoslits with the orientation along *b*‐axis show more obvious anisotropic photocurrents than those along *a*‐axis. As a result, the degree of anisotropy can reach a maximum value of 12.6. Similar polarization‐dependent responses are witnessed on the devices No. 2 and No. 3 as shown in Figure 5h,i, and the anisotropy ratio of each region is concluded in Figure 5j. We can conclude that only ReS_2_ region shows better performance on polarization‐sensitive detection than only WSe_2_ region, and the patterned heterojunctions further exhibit greater anisotropy than either only ReS_2_ region or unpatterned heterojunctions. Notably, comparisons among different devices are meaningless because the thicknesses, configurations, and dimensions are all different. For instance, shallow nanoslits with a thickness less than 30 nm in the top‐patterned No. 2 device (Figure S6, Supporting Information) weaken their contribution on polarization photodetection because of weak Mie resonances. Fortunately, the No. 1 device containing nanoslit arrays with both orientations provides a clear conclusion that the same direction of nanoslit, *b*‐axis, and polarization makes the largest anisotropy ratio. How the angle between nanoslit orientation and polarization direction determines the polarization‐sensitive photodetection can be understood from the schematic in Figure 5k and the reflection and field distribution measurements above. For the incident light polarized along the orientation of nanoslits, Mie resonant modes inside nanostructures can be excited with the electric field distributed uniformly, which is beneficial to the generation of electron–hole pairs. However, for the polarization direction perpendicular to the orientation of nanoslits, strong near‐field enhancements are only observed around the gap. This distribution feature leads to stronger scattering or reflection than the parallel polarization as well as weaker electric fields inside nanostructures to generate electron–hole pairs.

These findings demonstrate the capability of polarized light detection using patterned vdW heterostructures with precise design on crystal orientations and nanoslit orientations. To evaluate the photodetection performance of our devices with other WSe_2_‐ and ReS_2_‐related studies, important parameters are compared as shown in Table S1, Supporting Information. Our study shows the highest degree of anisotropy and competitive responsivity, on/off ratio, and rise/decay time compared with other studies. Through building interdigital electrode to divide each nanoslit array into independently controlled pixels, the proposed TMD‐based photodetectors with built‐in nanoslits, which are capable to engineer resonant wavelengths and polarization states, will be promising for polarization imaging^[^ ^48^ ^]^ and building ultracompact spectrometers.^[^ ^49^ ^]^

## 3 Conclusion

Our study demonstrates high‐performance polarization‐sensitive photodetection enabled by the anisotropic Mie resonances of built‐in ReS_2_ nanoslits. Through scattering measurement of single nanoslits and reflection characterization of nanoslit arrays, we demonstrated the strong anisotropic optical responses caused by the unique in‐plane anisotropy of ReS_2_ and polarization‐sensitive Mie resonances. Through the PL measurement, we found a coexistence mechanism of PL quenching arising from a typical type‐II band alignment and PL enhancement induced by the strong optical resonances. Nanoslit‐enhanced photodetectors with three configurations of patterned WSe_2_/ReS_2_ heterostructures were proposed to investigate how nanoslits influence the energy band remodeling and light trapping. The fabricated patterned WSe_2_/ReS_2_ heterostructures show high responsivity and fast response time owing to the efficient carrier separation and light trapping. Further, we quantitatively demonstrated the enhancement of photocurrent due to nanoslit arrays through the multi‐position excitation. Using linearly polarized laser to check the polarization‐sensitive photodetection of patterned hetero‐devices, an ultrahigh degree of anisotropy up to 12.6 was observed when the direction of nanoslit and polarization were both along *b*‐axis. These findings offer a new strategy for building polarization‐sensitive photodetectors in the future.

## 4 Experimental Section

### Sample Preparation and Device Fabrication

WSe_2_ and ReS_2_ flakes with varying thicknesses were prepared using mechanical exfoliation from bulk crystals (p‐type WSe_2_ and n‐type ReS_2_ purchased from HQ Graphene) through Nitto blue tapes and polydimethylsiloxane films. Subsequently, 2D flakes were transferred onto the designed locations on the Si/SiO_2_ substrate via the all‐dry transfer method. Next, Ti/Au (10/60 nm) electrodes were deposited on the designed area of heterostructures through a series of photolithography, e‐beam evaporation, and lift‐off processes. Last, the nanoslits were fabricated using FIB milling using the Zeiss Auriga FIB/SEM crossbeam system. A Ga‐ion beam was used in the milling process with a low dose (20 pA) at the 30 kV beam setting.

### Optical Spectroscopy and Numerical Simulations

The dark‐field scattering spectra of single nanoslits and reflectance spectra of nanoslit arrays were measured using an optical microscope (BX53, Olympus) integrated with a spectrograph and a CCD camera (Princeton Instruments). An objective (100×, NA = 0.80) was used for both white light illuminations and scattering/reflection collections, and the scattering (reflection) signal was collected using dark‐field (bright‐field) mode. PL spectra were collected using a confocal Raman microscope equipped with a 532 nm laser incidence via a 100× objective lens (NA = 0.9). The scattering/reflection spectra and near‐field distributions were simulated using the finite‐difference time‐domain method. The refractive index of ReS_2_ was obtained from relevant literatures and fitted using multi‐coefficient model to expand the wavelength range. The total‐field scattered‐field normal incident light with a top planar detector was applied to collect the backward scattering, and a cross‐sectional planar detector was used to obtain the electric and magnetic near‐field profiles.

### Optoelectronic Characterizations

For the entire devices, the photoresponse measurements were completed in a probe station integrated with a Keithley 4200 semiconductor parameter analyzer. Laser sources with the wavelengths of 405 and 532 nm were used, and the spot diameter was 5 mm covering the entire devices. Switch on or off of light was controlled by a chopper, and the rise and decay time were measured using an oscilloscope (Tektronix DPO 4102B). For the single‐point measurement, devices were placed under an optical microscope integrated with a 532 nm laser and a tunable polarizer. The laser beam was focused to 10 µm, and the photocurrents were measured through movable probes mounted around the sample stage. Small tilted angle of probes was used to adapt the focal length of objective.

## Conflict of Interest

The authors declare no conflict of interest.

## Supporting information

Supporting Information

## Data Availability

The data that support the findings of this study are available from the corresponding author upon reasonable request.
