# Supplementary material for: Van der Waals Heterostructures With Built‐In Mie Resonances For Polarization‐Sensitive Photodetection
Source: Adv Sci (Weinh). 2023 Jan 22;10(9):2207022. doi: 10.1002/advs.202207022 (PMC10037953; doi:10.1002/advs.202207022)
Supplement: Supplementary file 1 — Supporting Information [file ADVS-10-2207022-s001.pdf]

ADVANCED SCIENCE
Open Access

Supporting Information

for *Adv. Sci.*, DOI 10.1002/advs.202207022

Van der Waals Heterostructures With Built-In Mie Resonances For Polarization-Sensitive Photodetection

Jiahao Yan, Xinzhu Yang, Xinyue Liu, Chun Du, Fei Qin, Mengmeng Yang, Zhaoqiang Zheng* and Jingbo Li**

Supporting Information

van der Waals heterostructures with built-in Mie resonances for polarization-sensitive photodetection

Jiahao Yan[1]*, Xinzhu Yang[1], Xinyue Liu[1], Chun Du[4], Fei Qin[4], Mengmeng Yang[2], Zhaoqiang Zheng[2]*, & Jingbo Li[3]*.

1 Institute of Nanophotonics, Jinan University, Guangzhou 511443, China

2 Guangdong Provincial Key Laboratory of Information Photonics Technology, School of Materials and Energy, Guangdong University of Technology, Guangzhou 510006, China

3 Institute of Semiconductors, South China Normal University, Guangzhou 510631, China

4 Guangdong Provincial Key Laboratory of Optical Fiber Sensing and Communications, Institute of Photonics Technology, Jinan University, Guangzhou 511443, China

*Corresponding author: jhyan@jnu.edu.cn & zhengzhq5@mail2.sysu.edu.cn & jbli@semi.ac.cn

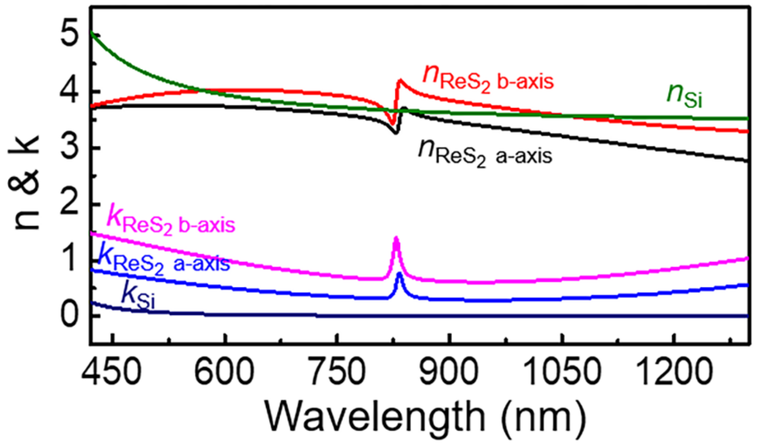

Figure S1. Refractive indices (*n*) and extinction coefficients (*k*) of Si and ReS_2 along two orthogonal axes. Related data [1] have been utilized, and further fitting using multi-coefficient model have been done to expand the wavelength ranges.

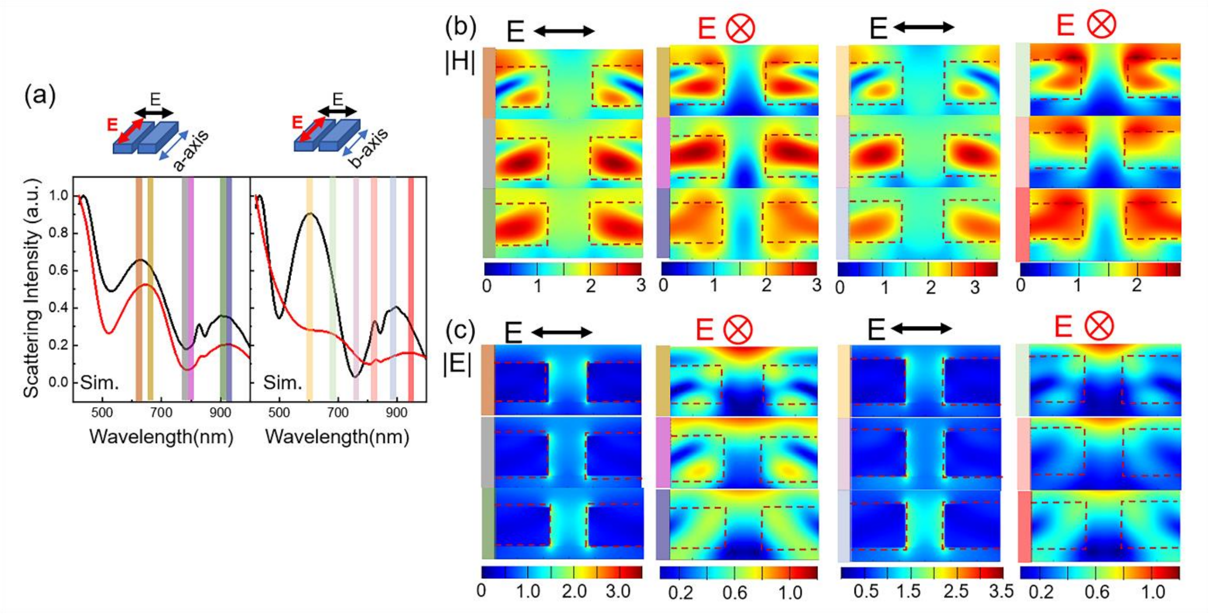

Figure S2. (a) Simulated scattering spectra of nanoslits under linearly polarized light. Embedded diagrams show the directions of polarization and nanoslit. Vertical bars in simulated spectra indicate the locations where the near-field profiles collected. (b, c) Near-field magnetic (b) and electric (c) field distributions of ReS_2 nanoslits at different wavelengths marked by the color correspondence.

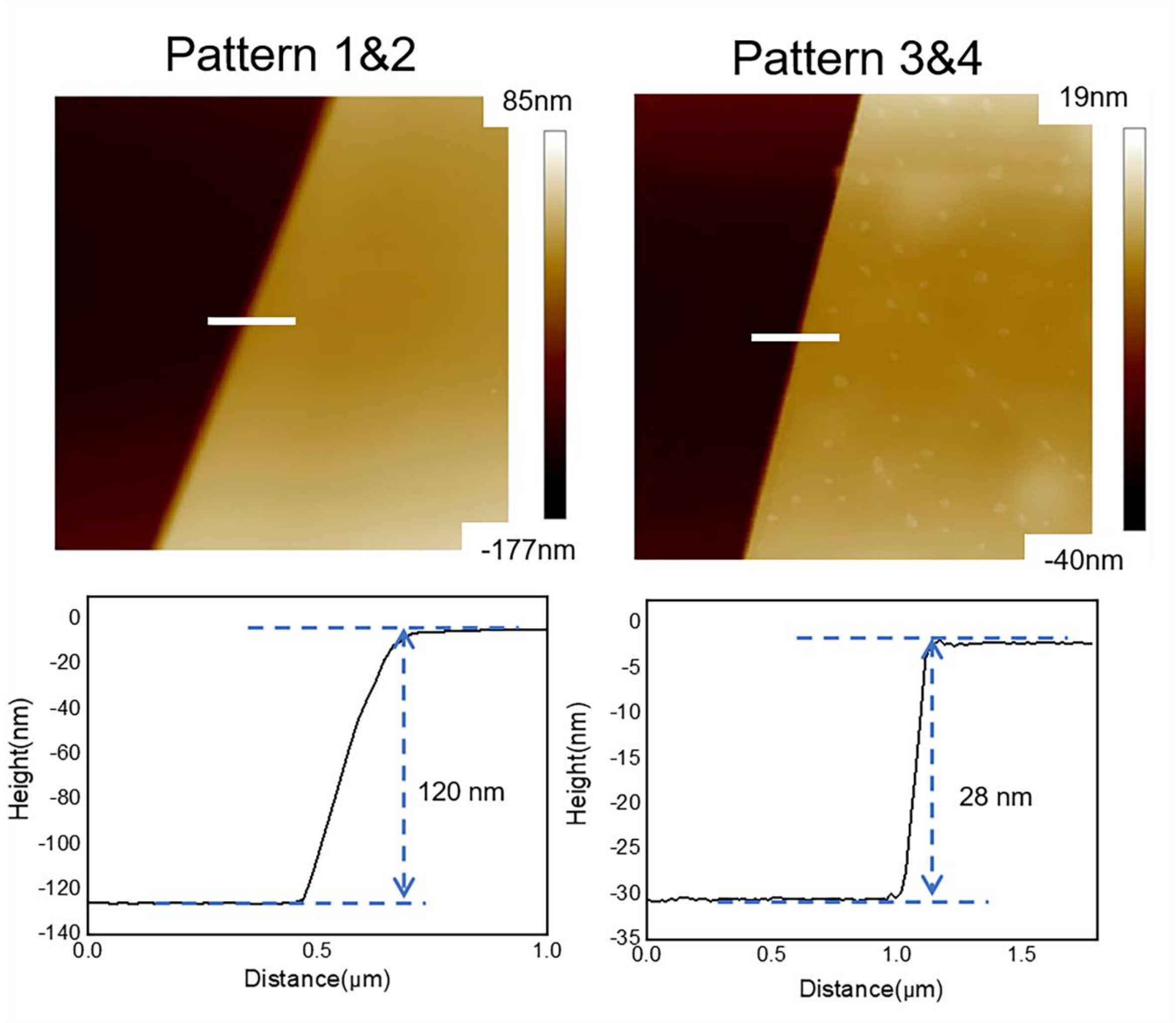

Figure S3. AFM images of thick ReS_2 with Pattern 1&2 and thin ReS_2 with Pattern 3&4, and the corresponding height profiles.

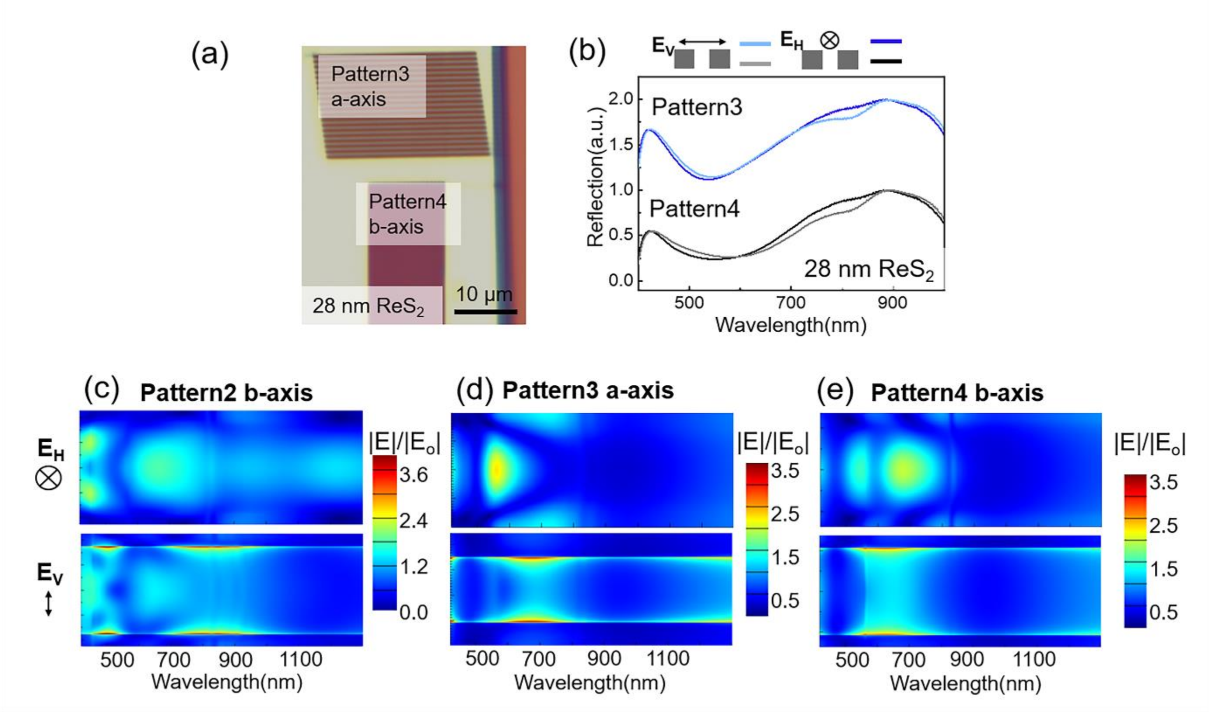

Figure S4. (a) The optical image of ReS_2 (28 nm) nanoslits. The orientation of nanoslits is along a-axis (b-axis) in Pattern 3 (4). (b) Measured reflection spectra of nanoslit arrays on 28 nm ReS_2 under horizontal (E_H) or vertical (E_V) polarization. (c) Wavelength-dependent electric field distributions along the top surface of the nanoslit (Pattern 2) patterned on 120 nm ReS_2 under two polarization directions. (d, e) Wavelength-dependent electric field distributions along the top surface of the nanoslit arrays (Pattern 3 & 4) patterned on 28 nm ReS_2 under two polarization directions.

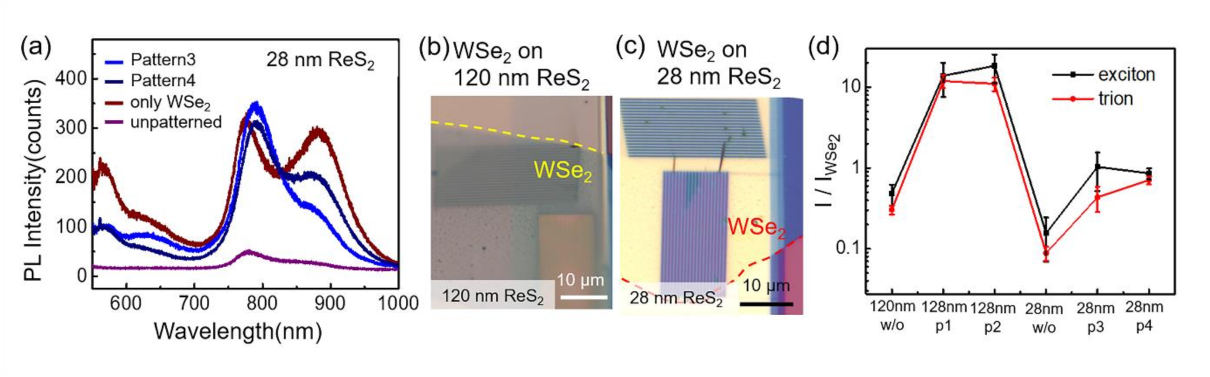

Figure S5. (a) PL spectra of few-layer WSe_2 on the Si/SiO_2 substrate, unpatterned ReS_2 (28 nm) and patterned ReS_2 (Pattern 3 and 4). (b, c) Optical images of patterned thick ReS_2 (a) and thin ReS_2 (b) with transferred WSe_2 few-layers marked by dashed lines. (d) Averaged PL intensity ratios (I/I_{WSe2}) and the corresponding standard deviations at each region. Two typical excitonic wavelengths (exciton and trion) are considered. P1-4 are abbreviations for Pattern 1-4, and w/o means the unpatterned heterostructure.

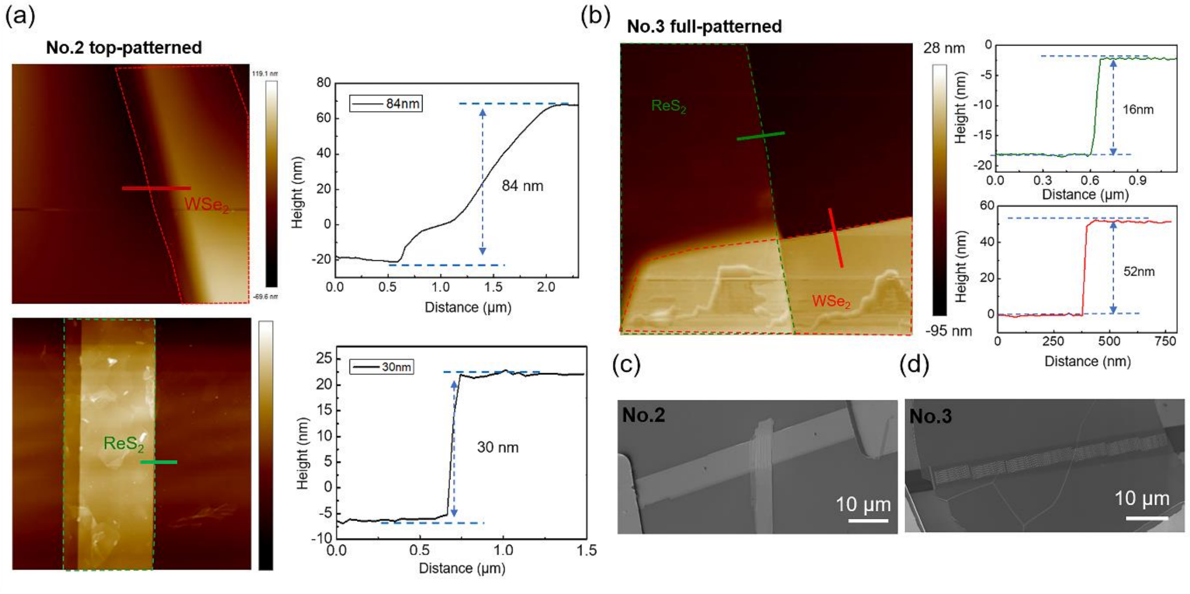

Figure S6. (a, b) AFM images of heterostructures (No. 2&3) and the corresponding height profiles. The red (green) line indicates the measured region of WSe_2 (ReS_2). (c, d) SEM images of patterned heterostructures (No. 2&3).

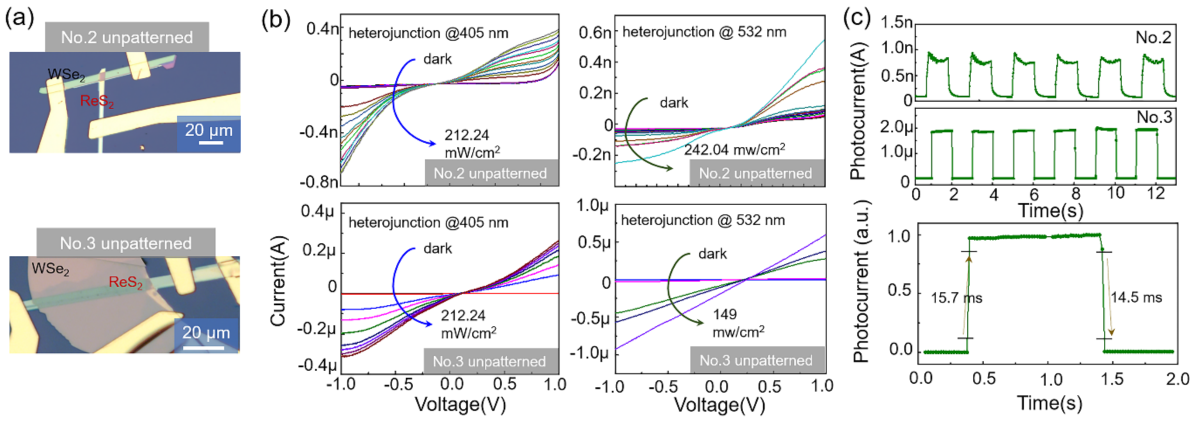

Figure S7. (a) Optical images of WS_2/ReS_2 hetero-devices named as No.2&3 before patterning. (b) I-V characteristics of No.2 and No.3 photodetectors under light irradiation (405 nm and 532 nm) with various intensities. (c) The photoswitching characteristics of two hetero-devices under 532 incidence, and a temporal photoresponse of a single photoswitching cycle showing the best performance.

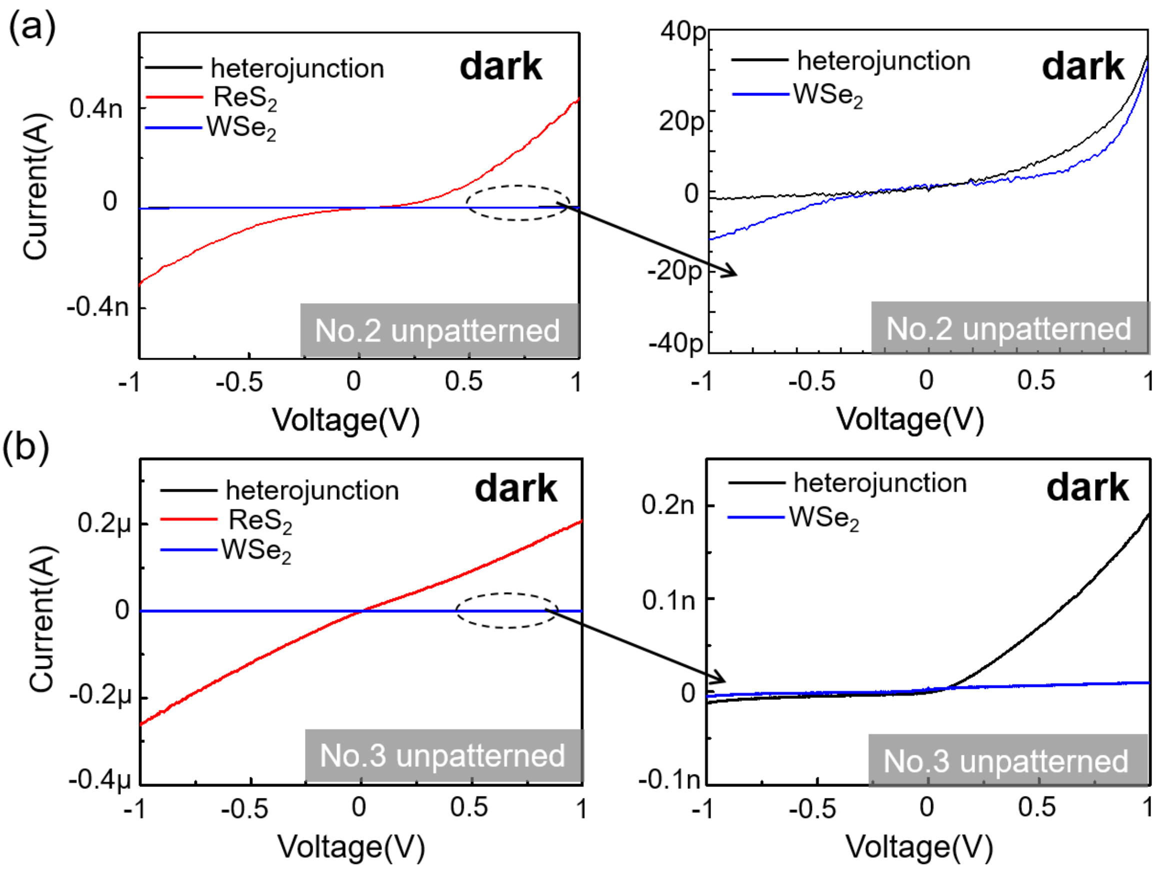

Figure S8. I-V characteristics of the unpatterned devices No.2&3 without illumination showing the rectification behaviors of heterojunction (black), only ReS_2 (red), and only WSe_2 (blue) regions.

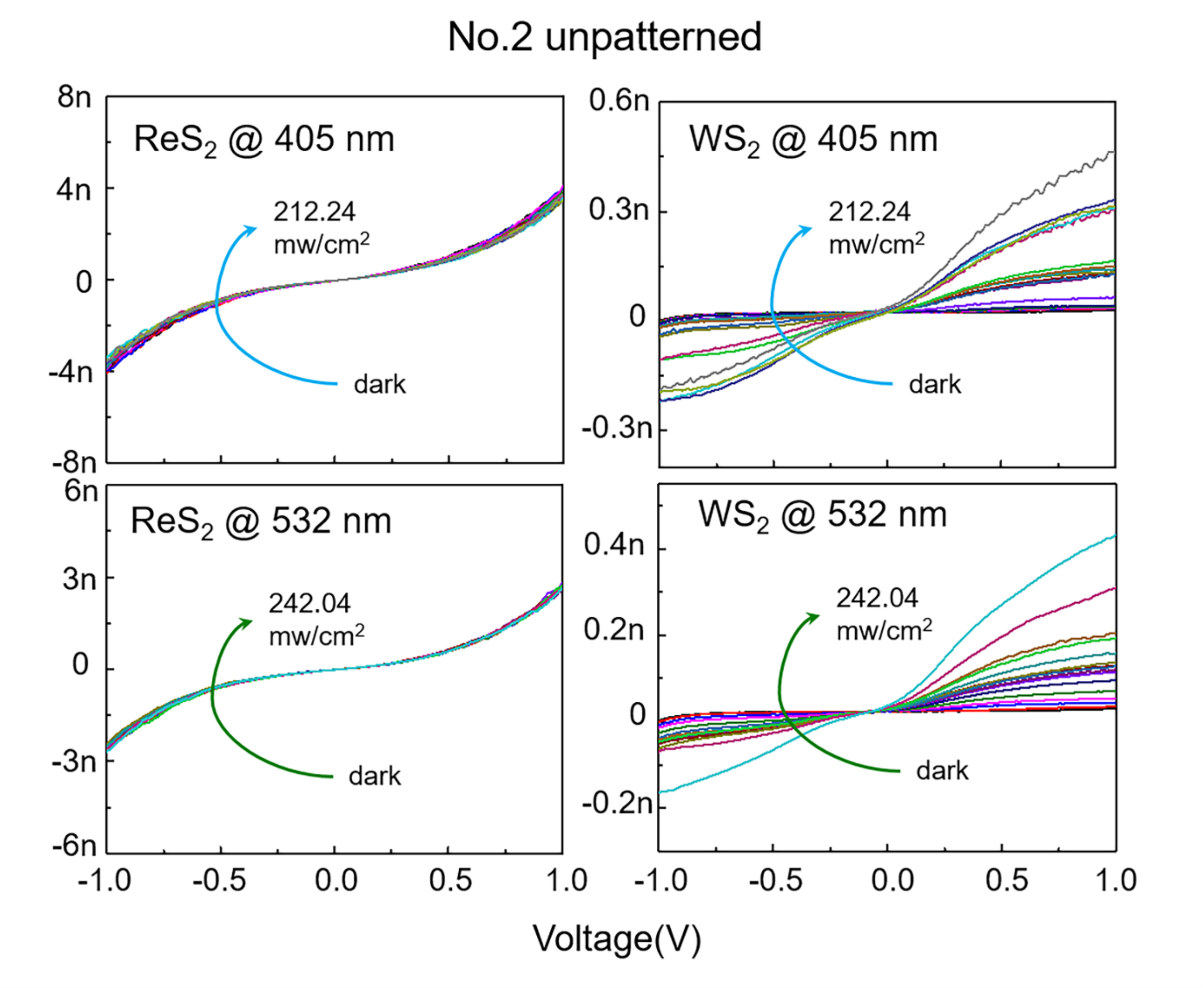

Figure S9. I-V characteristics of only ReS_2 or only WSe_2 region of the device No.2 unpatterned under light irradiation (405 and 532 nm) with various intensities.

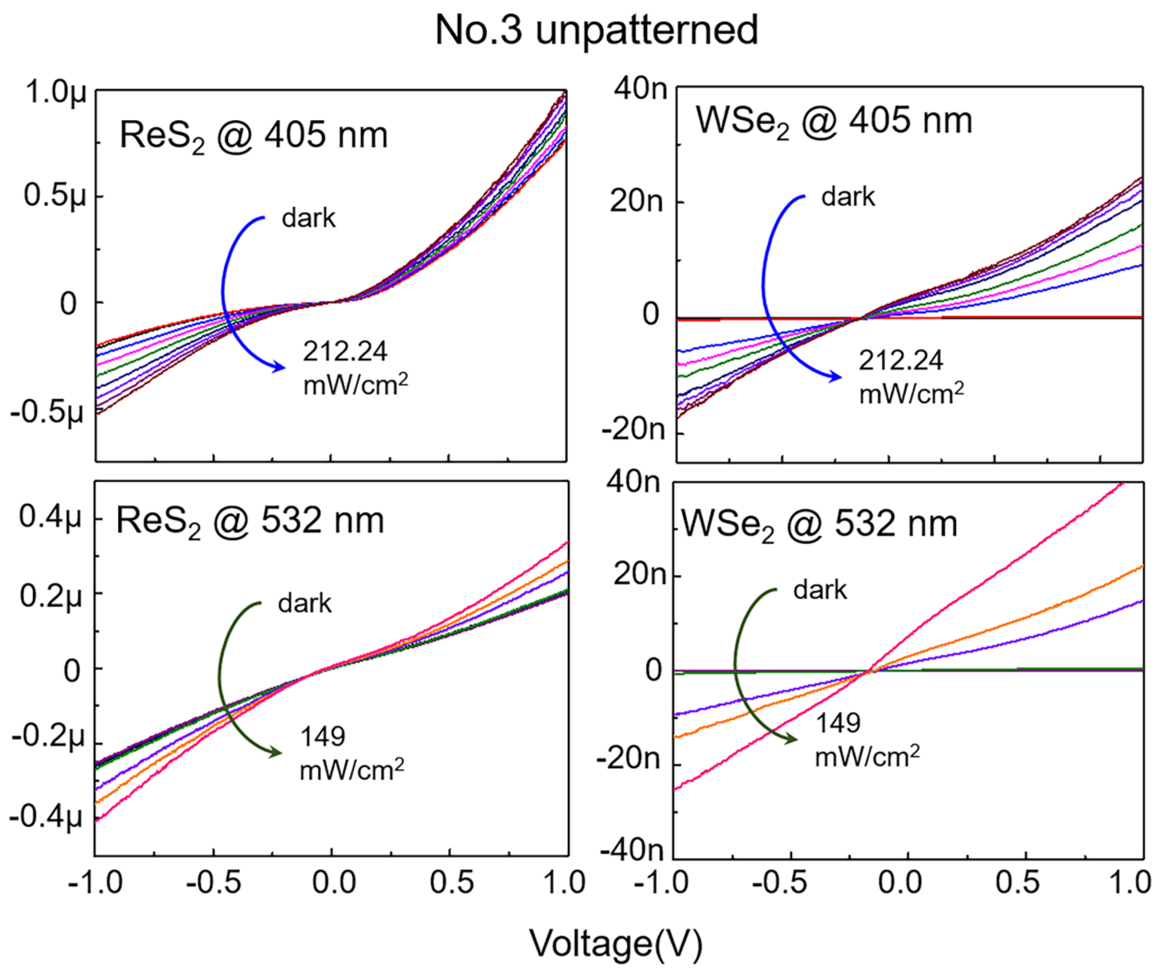

Figure S10. I-V characteristics of only ReS_2 or only WSe_2 region of the device No.3 unpatterned under light irradiation (405 and 532 nm) with various intensities.

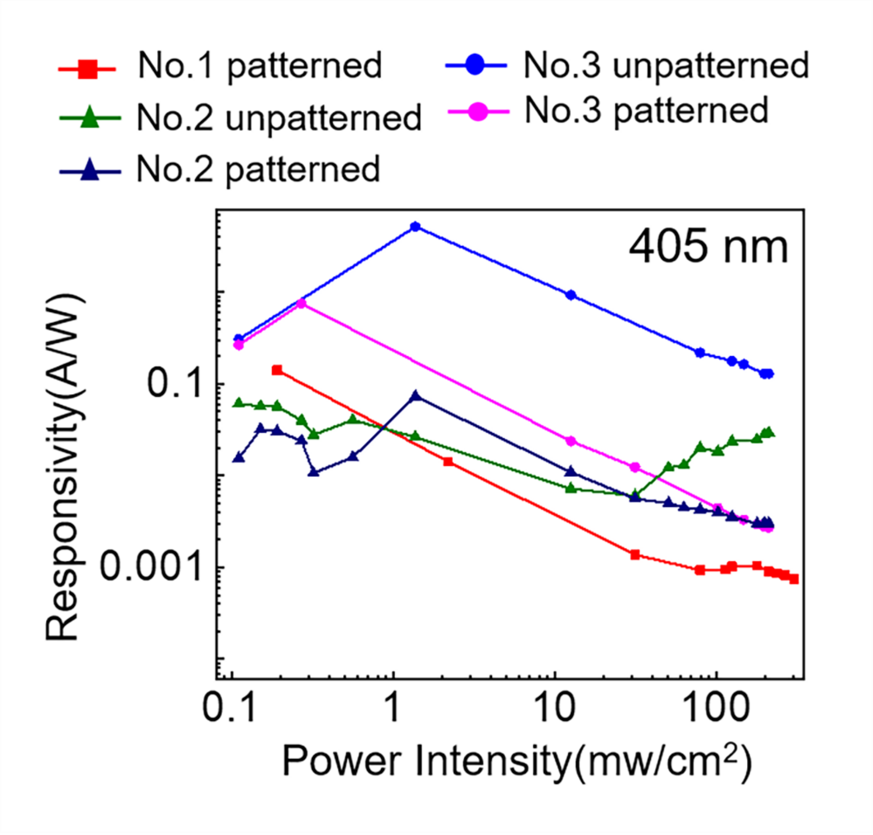

Figure S11. Responsivity under 405 nm irradiation of unpatterned and patterned heterostructures calculated from the photocurrent measurements.

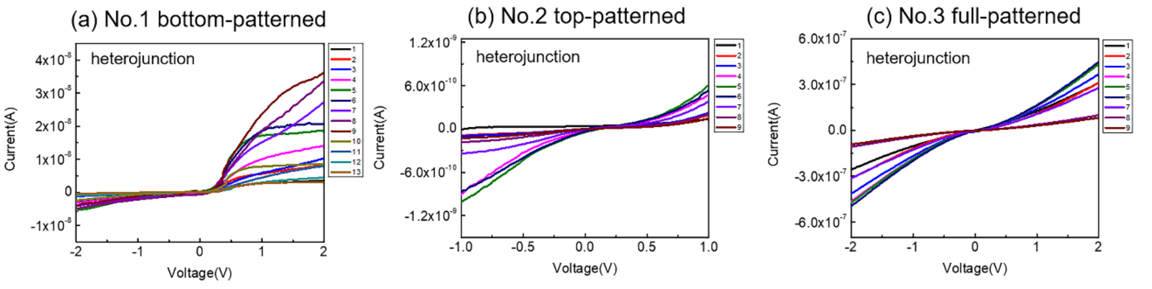

Figure S12. Location-dependent I−V characteristics of the patterned devices No.1-3 under 532 nm laser excitation.

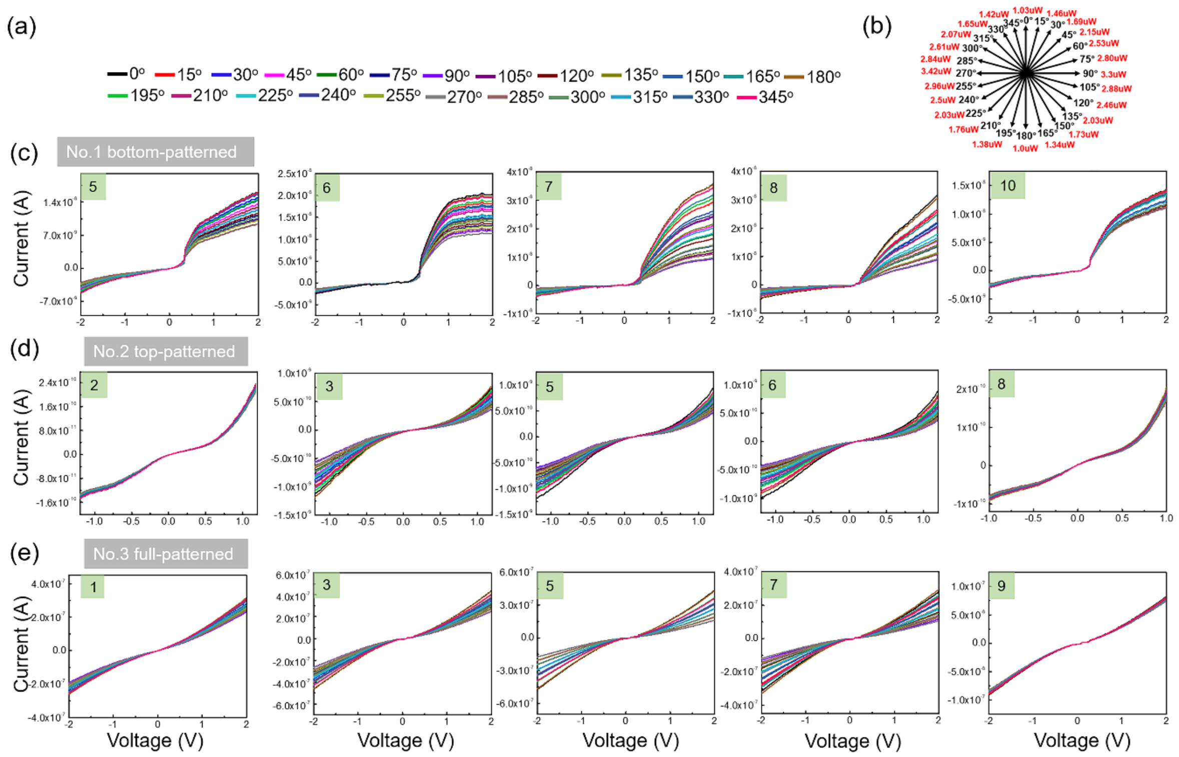

Figure S13. (a) Labels of polarization directions. (b) A schematic showing the laser power of linearly polarized light with varied angles. (c-e) Polarization-dependent I−V characteristics of the patterned devices No.1-3 under 532 nm laser excitation. The results in Fig. 5g-i were calculated using the values of photocurrents in (c-e) divided by the laser power in (b).

Devices	Responsivity	On/Off Ratio	Rise/Decay Time	Anisotropy Ratio	Reference
WSe_2/ReS_2 (unpatterned)	140 A/W	10^2	15.7/14.5 ms	4.3	This work
WSe_2/ReS_2 (patterned)	27 A/W	10^4	3.7/3.7 ms	12.6	This work
WSe_2/ReS_2	3 A/W	10^5	5 μs	——	2
ReS_2/graphene/WSe_2	1.02 A/W	——	44 ms	1.33	3
WS_2/ReS_2	1.85 mA/W	10^2	1.30/0.85 ms	1.9	4
ReS_2	10^3 A/W	10^5	——	4	5
ReS_2	12 A/W	10^6	——	——	6
ReS_2	88600 A/W	10^8	>100 s	——	7
ReS_2	4 A/W	10^4	20 μs	——	8
ReS_2	16.14 A/W	10^6	>100 s	——	9
suspended ReS_2	0.22 A/W	10^5	83.5/325.3 μs	1.4	10
$MoTe_2/ReS_2$	0.54 A/W	10^4	2.4/3.0 μs	1.03	11
WSe_2 p-n junction	0.8 A/W	10^6	136/39 μs	——	12
WSe_2-WSe_2 homojunction	11.2 mA/W	——	180 μs	——	13
WSe_2 p-i-n junction	0.1 A/W	10^5	264/552 ns	——	14
WSe_2	2.31 A/W	5.82×10^4	——	——	15
WSe_2	0.92 A/W	——	0.9 s	——	16
WSe_2/Ag nanoprism	1.5 μA/W	——	——	1.6	17

Table S1. Performance comparison between photodetectors based on WSe_2-related, ReS_2-related, and WSe_2/ReS_2 heterostructures.[2-17]

References

1. Wang, Y. Y. *et al.* In-plane optical anisotropu in ReS_2 flakes determined by angle-resolved polarized optical contrast spectroscopy. *Nanoscale* **11**, 20199-20205 (2019).
2. Varghese, A. *et al.* Near-direct bandgap WSe_2/ReS_2 type-II pn heterojunction for enhanced ultrafast photodetection and high-performance photovoltaics. *Nano Lett.* **20**, 1707-1717 (2020).
3. Wang, Z. *et al.* Broadband photodetector based on ReS_2/graphene/WSe_2 heterostructure. *Nanotechnology* **32**, 465201 (2021).
4. Tang, Y. *et al.* Distinctive interfacial charge behavior and versatile photoresponse

performance in isotropic/anisotropic WS_2/ReS_2 heterojunctions. *ACS Appl. Mater. Inter.* **12**, 53475-53483 (2020).

5. Liu, F. *et al.* Highly sensitive detection of polarized light using anisotropic 2D ReS_2. *Adv. Funct. Mater.* **26**, 1169-1177 (2016).
6. Li, X. *et al.* Controlled growth of large-area anisotropic ReS_2 atomic layer and its photodetector application. *Nanoscale* **8**, 18956-18962 (2016).
7. Liu, E. *et al.* High responsivity phototransistors based on few-layer ReS_2 for weak signal detection. *Adv. Funct. Mater.* **26**, 1938-1944 (2016).
8. Thakar, K. *et al.* Multilayer ReS_2 photodetectors with gate tunability for high responsivity and high-speed applications. *ACS Appl. Mater. Inter.* **10**, 36512-36522 (2018).
9. Zhang, E. *et al.* ReS_2-based field-effect transistors and photodetectors. *Adv. Funct. Mater.* **25**, 4076-4082 (2015).
10. Zhong, J. *et al.* Direct observation of enhanced performance in suspended ReS_2 photodetectors. *Opt. Exp.* **29**, 3567-3574 (2021).
11. Ahn, J. *et al.* 2D $MoTe_2$/ReS_2 van der Waals heterostructure for high-performance and linear polarization-sensitive photodetector. *ACS Photon.* **8**, 2650-2658 (2021).
12. Chen, J. *et al.* High-performance WSe_2 photodetector based on a laser-induced p–n junction. *ACS Appl. Mater. Inter.* **11**, 43330-43336 (2019).
13. Tan, C. *et al.* A self-powered photovoltaic photodetector based on a lateral WSe_2-WSe_2 homojunction. *ACS Appl. Mater. Inter.* **12**, 44934-44942 (2020).
14. Zhang, Y. *et al.* An ultrafast WSe_2 photodiode based on a lateral pin homojunction. *ACS Nano* **15**, 4405-4415 (2021).
15. Zhou, C. *et al.* Self-driven metal–semiconductor–metal WSe_2 photodetector with asymmetric contact geometries. *Adv. Funct. Mater.* **28**, 1802954 (2018).
16. Zheng, Z. *et al.* Flexible, transparent and ultra-broadband photodetector based on large-area WSe_2 film for wearable devices. *Nanotechnology* **27**, 225501 (2016).
17. Guskov, A., Lavrov, S. & Galiev, R. Polarization sensitive photodetectors based on two-dimensional WSe_2. *Nanomaterials* **12**, 1854 (2022).
